# The Notch system during pubertal development of the bovine mammary gland

**DOI:** 10.1038/s41598-019-45406-6

**Published:** 2019-06-20

**Authors:** Nadia Bonadeo, Damasia Becu-Villalobos, Carolina Cristina, Isabel M. Lacau-Mengido

**Affiliations:** 1 Centro de Investigaciones Básicas y Aplicadas, Centro de Investigaciones y Transferencia del Noroeste de la Provincia de Buenos Aires, Monteagudo 2772, Pergamino 2700, Buenos Aires, Argentina; 2 Instituto de Biología y Medicina Experimental, IBYME-CONICET, Vuelta de Obligado 2490, Ciudad Autónoma de Buenos Aires, 1428 Argentina

**Keywords:** Cell signalling, Stem cells

## Abstract

The development of the mammary gland of cows during pre-weaning and puberty will condition its future productive capacity and warrants special study. In this respect, Notch signaling regulates tissue development and fate by modifying cell proliferation and differentiation and has been involved in stem cell maintenance, but has not been extensively studied in the developing mammary glands in cows. We therefore investigated Notch receptor expression and localization, as well as the expression of Notch ligands and target genes in the mammary gland of Holstein heifers in pre- and post-pubertal stages. Notch receptors 1 to 4 were detected by immunohistochemistry in the parenchyma and stroma of the developing gland. The subcellular localization of the four receptors was predominantly cytoplasmic except for NOTCH4, which was mostly nuclear. The membrane and the active intracellular domains of NOTCH paralogues were identified by western blot. NOTCH1 and NOTCH2 active domains increased during pubertal stages while NOTCH3 and NOTCH4 active domains decreased, suggesting strikingly different involvement of NOTCH paralogues in bovine mammary gland development and differentiation. The mRNA expression levels of the target genes *HEY1* and *HEY2* increased during peri-puberty whereas no variation of *HES1* mRNA levels was observed. The mRNA levels of the Notch ligands *JAGGED1* and *DELTA1* also increased gradually during development. In conclusion, Notch signaling system dynamically varies throughout the development of the mammary gland during puberty pointing to specific time involvement of each component.

## Introduction

It has been stated that the future milk yield potential of cows depends on the optimum development of the mammary parenchyma that is established during growth, starting as early as the first weeks of life^1,2^. Before and around puberty the gland grows allometrically (faster than the rest of the body), mainly in response of increasing estradiol and IGF1 levels^3^. An arborescent ductal network of epithelial cells grows from the nipple to the interior of the gland into a “fat pad” consisting of stromal tissue composed by extracellular matrix, fibroblasts, adipocytes and capillaries. On the other hand, the parenchymal tissue of the gland is composed of epithelial structures and will be responsible for production and transport of the milk. The mammary epithelium is composed of two layers of epithelial cells: luminal cells of cubical shape that line the hollow lumens of the ducts, surrounded by flat myoepithelial cells that have a contractile function. As the gland matures, branching and ductal elongation occur in response to endocrine and paracrine stimuli^4,5^. It has been shown that nutrition plays an important role in regulating the growth of the bovine mammary gland in the early stages of development, specially pre weaning^6^. Efforts are now being directed to elucidate genes involved in this process^7,8^. After puberty, the mammary gland undergoes repeated cycles of development and involution coordinated with pregnancy and lactation cycles. This process of growth and regression points to a hierarchy of stem and progenitor cells that are able to regenerate specialized populations of mammary epithelial and myoepithelial cells. Over the last decades, evidence for the existence of mammary stem cells (MaSC), capable of self-renewing and generating multiple mammary cell types has accumulated mainly in mice and humans, but also in bovines and other farm animal species^9–12^. Recently a cell lineage hierarchy has been proposed in the bovine mammary gland based on different stem cell markers^13^, and differential evolution of progenitor cell populations during lactation has been described^14^.

Notch pathway is conserved from flies to mammals. It regulates numerous cellular processes like cellular proliferation, differentiation, and apoptosis and has a relevant role in stem cell maintenance^15,16^. Notch signaling includes cell to cell interactions by means of receptors and ligands, anchored on cell membranes of neighboring cells. Mammalian cells have four Notch receptors (NOTCH1–4) and at least five DELTA/JAGGED different ligands. Notch receptors are coded by a single precursor that is activated by post-transductional proteolytic cleavage resulting in a heterodimer, where the extracellular domain is non covalently bound to the transmembrane/intracellular domain^17^. They are protein heterodimers with a single pass type I transmembrane/intracellular domain and an extracellular domain involved in ligand binding. The Notch intracellular domain (NICD) contains nuclear localization signals and the transcriptional activation domain^18^. The intracellular transduction of Notch signaling is remarkably simple, with no secondary messengers involved. Receptor interaction with the ligand triggers successive cleavages of the Notch receptor, the last involving the intracellular γ-secretase protein complex, which finally releases the NICD. This active domain translocates to the nucleus where it interacts with transcription factors to activate specific genes involved in proliferation, differentiation and survival^19^. The binding and function of NOTCH on DNA is a rapid and dynamic process controlled by phosphorylation, ubiquitination and subsequent proteasomal degradation, which shuts off the pathway. In the absence of NICD, the transcription factor RBPJ (Recombining binding protein suppressor of hairless) forms complexes with a variety of corepressors to suppress the transcription of Notch target genes^20,21^. Many Notch target genes have been identified. Among them are the *HES* (hairy enhancer of split) and *HEY* (Hairy/enhancer-of-split related with YRPW motif protein) families, *cyclin D1, Akt, c-myc, COX-2* (cyclooxygenase-2) and *mTOR* (mammalian target of rapamycin)^22^.

The Notch signaling system has been shown to maintain precursor cells by balancing cellular proliferation, cell fate and differentiation in several tissues, such as brain, muscle, intestine and hematopoietic cells^23–27^. Notch pathway activity has been associated with stem cells in many tissues, and therefore it has been used to trace lineages in some tissues like the intestine and the mammary gland^9,28,29^. Recently, increasing amount of work has approached Notch signaling in the mammary gland to decipher the lineage hierarchies that contribute to mammary development, and understand the morphogenesis of the gland. Furthermore new insights have linked the Notch system to the cellular origins of mammary tumors^15,30,31^. In cows, however, a paucity of studies have been performed on Notch signaling in the mammary gland^13,32^. During development, all four Notch receptor paralogues, NOTCH1–4, have been shown to be expressed in the mouse and the human mammary gland^33,34^, however no reports are yet found on Notch signaling in the pubertal bovine mammary gland. With the perspective of understanding all insights of the regulation of mammary development during puberty which will impact on performance of the future gland in adulthood, we investigated the expression and localization of the four Notch receptor paralogues, as well as the expression of two ligands and three target genes in the bovine mammary gland before, during and after puberty onset.

## Results

**Figure 1:**
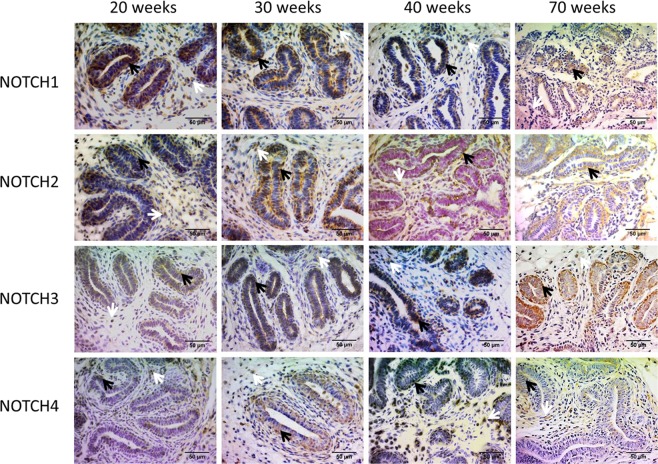
Representative images of immunohistochemical labeling of mammary gland biopsy samples, for the four NOTCH paralogues at 20, 30, 40 and 70 weeks of age. Notch labelling is evidenced in stromal cells (white arrows) and in epithelial and myoepithelial cells of the parenchyma (black arrows).

We performed immunohistochemical labeling of the four Notch receptors in paraffin sections of biopsies of the mammary gland of Holstein heifers, taken at 20, 30, 40 and 70 weeks of age (representative images are shown in Fig. 1). Notch labeling was positive for the four paralogues at all ages investigated. NOTCH1 was mostly found in epithelial and myoepithelial cells of the parenchyma though its presence was also evidenced in stromal cells. The label was identified at membrane, cytoplasmic and nuclear levels. NOTCH2 was evidenced in parenchymal and stromal cells at cytoplasmic and nuclear levels, and it was predominantly found in the cytoplasm of epithelial and myoepithelial cells. NOTCH3 was identified mostly in the cytoplasm of epithelial and myoepithelial cells and, with lesser intensity, in some stromal cells. The label of NOTCH4 was stronger in myoepithelial cells, and was predominantly nuclear, but it was also evidenced, with lesser intensity, at cytoplasmic level. No particular age pattern of labeling could be evidenced with this technique for any of the four Notch receptor paralogues. In summary, the four Notch paralogues are present in both compartments, parenchyma and stroma, with the exception of NOTCH3 that is rarely expressed in stromal cells. Furthermore, NOTCH1–3 are predominantly expressed in the cytoplasm while NOTCH4 is almost exclusively expressed in the nucleus.

**Figure 2:**
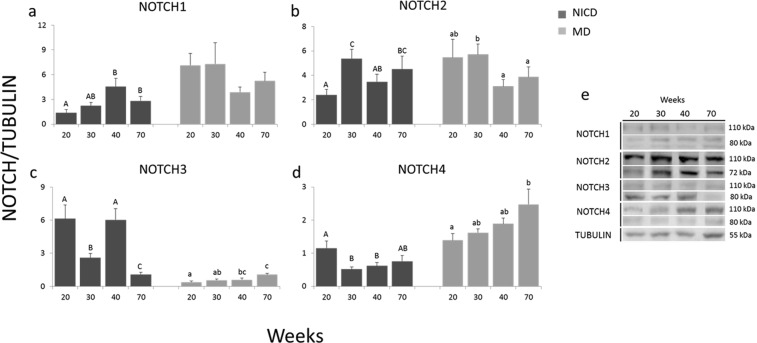
Intracellular (NICD, black bars) and membrane (MD, grey bars) domains of NOTCH1–4 were determined by Western blot in mammary glands of heifers aged 20, 30, 40 and 70 weeks, and were expressed in relation to tubulin expression (n = 6, 10, 10, 10 **(a)**); (n = 7, 10, 10, 9 **(b)**); (n = 10, 10, 10, 7 **(c)**); (n = 7, 7, 7, 6 **(d)**). For this and the following figures, lines on the top of the bars represent 1 SE. Different capital letters mean statistical differences (P < 0.05) between ages for NICD, and lower case letters for MD. Representative Western blots showing the active NICD (80 or 72 kDa) and membrane domains (110 kDa) of NOTCH 1–4 receptors in the four ages studied are shown **(e)**. Full-lenght blots are shown in Supplementary Fig. S1.

When Western blots were performed for each Notch receptor, two bands were identified, one at 80 KDa (or 72 KDa depending on the paralogue) which corresponds to the active intracellular domain (NICD) and the other one at 110 KDa, corresponding to the membrane receptor. In Fig. 2, mean band intensities, related to tubulin, are graphed for all ages studied, and representative images of the bands are shown. Active NOTCH1 expression increased significantly with age, and was higher during the peripubertal (40 weeks) and postpubertal periods (70 weeks) than at prepuberty (20 weeks), in contrast to the membrane receptor which did not show significant differences between ages. NOTCH2 intracellular domain expression increased at 30 weeks during early puberty and then decreased to prepubertal levels at 40 weeks, and at 70 weeks remained higher than at early prepuberty, whereas the membrane domain was higher during early puberty at 30 weeks of age than at 40 and 70 weeks. NOTCH3 the active domain was lower at 30 and 70 weeks compared to 20 and 40 weeks of age, whereas the membrane receptor was higher at 70 weeks compared to 20 and 30 weeks of age. Active NOTCH4 expression was higher at 20 weeks of age and then decreased, while the membrane receptor increased with age and was lower at 20 weeks (prepuberty) compared with 70 weeks (postpuberty).

**Figure 3:**
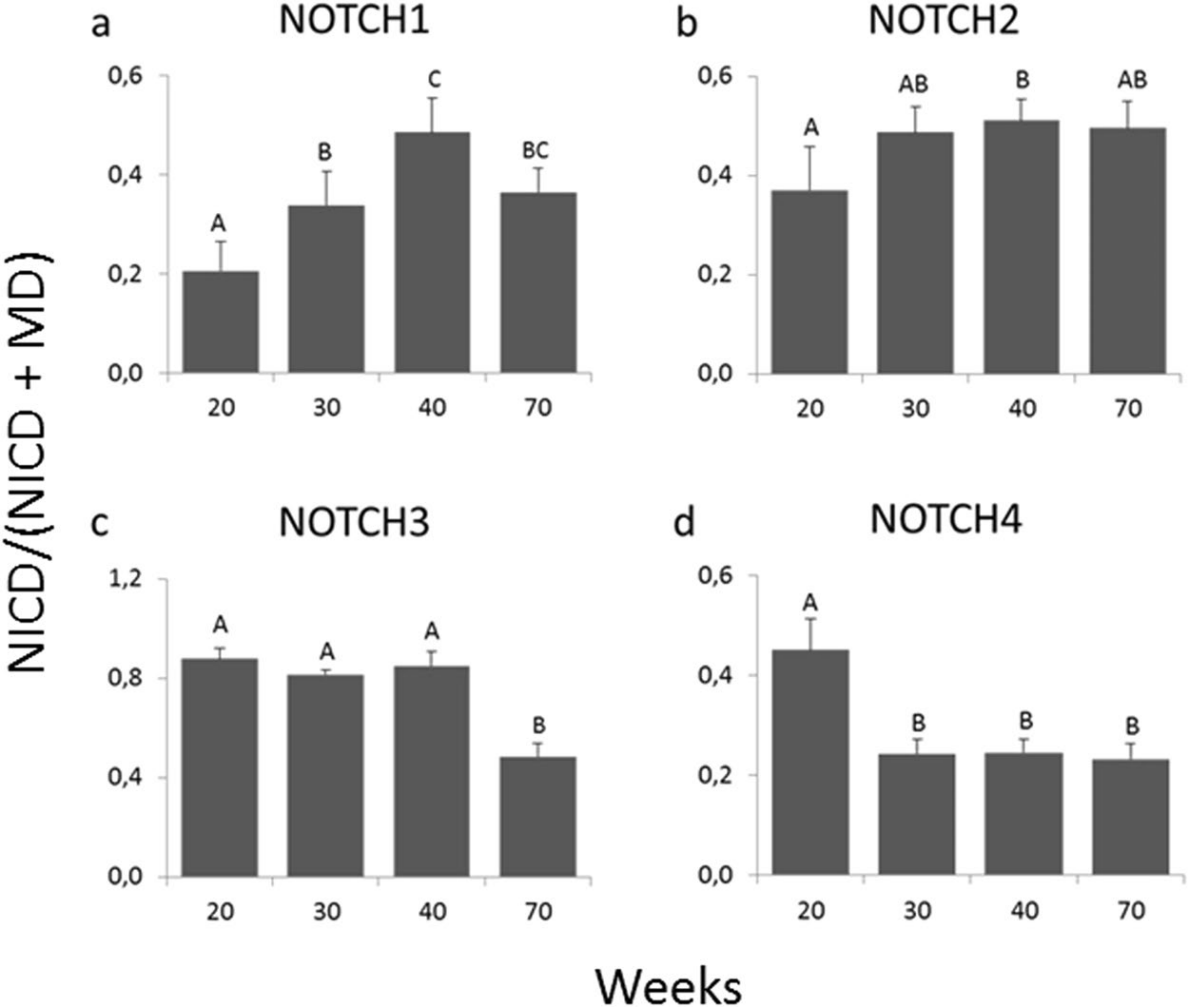
Relative proportion of the active domain over total receptor (NICD/(NICD + MD)) for NOTCH1–4 in mammary glands of heifers at 20, 30, 40 and 70 weeks of age (n = 6, 10, 10, 10 **(a)**); (n = 7, 10, 10, 9 **(b)**); (n = 10, 10, 10, 7 **(c)**); (n = 7, 7, 7 6 **(d)**). Different letters on the top of the bars denote statistical differences between ages.

In Fig. 3 we analyzed the developmental pattern of the relative proportion of the NICD domain relative to the total NOTCH levels, that is the active and membrane receptor (NICD/(NICD + MD), i.e. 80 KDa/(80 + 110 KDa) or 72KDa/(72 + 110KDa)). We observed that the relative NICD for NOTCH1 (relative NICD1) increased with age, being higher at 30 than at 20 weeks of age, and higher at 40 than at 20 and 30 weeks. At 70 weeks it remained higher than at 20 weeks, but was not different from 30 and 40 weeks. Relative NICD2, also increased with age, and was higher at 40 than at 20 weeks. No differences were observed for 30 and 70 weeks of age. For NOTCH3 and 4, the relative NICD decreased with age. Relative NICD3 was lower at 70 weeks compared with 20, 30 and 40 weeks and relative NICD4 was lower at 30, 40 and 70 weeks than at 20 weeks. These results reveal striking differences in the development of each active paralogue: while active NOTCH1 and 2 increase with pubertal development, NOTCH3 and 4 decrease.

**Figure 4:**
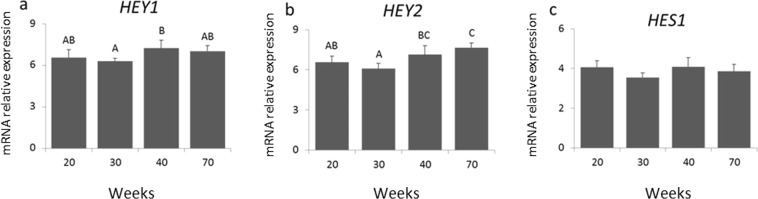
Differential gene expression of Notch target genes *HEY1, HEY2* and *HES1*, normalized to *GAPDH*, were determined by qRT-PCR in mammary biopsies from heifers aged 20, 30, 40 and 70 weeks (n = 4, 10, 7, 9 (**a)**); (n = 3, 8, 7, 8 **(b)**); (n = 3, 9, 7, 10 **(c)**). Different letters indicate significant differences (p ≤ 0.05) between ages.

**Figure 5:**
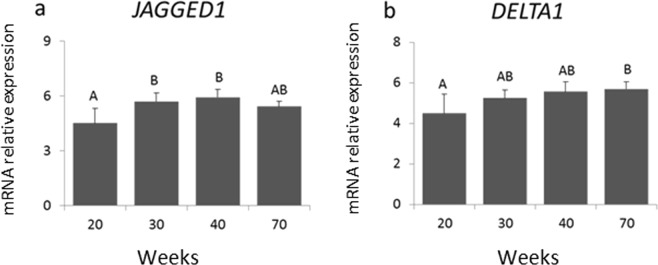
mRNA expression determined by qRT-PCR from Notch ligands *JAGGED1* and *DELTA1*, normalized to *GAPDH*, in the mammary glands from heifers aged 20, 30, 40 and 70 weeks (n = 5, 8, 9, 10 **(a)**); (n = 3, 8, 7, 7 **(b)**). Different letters on the top of the bars indicate statistical differences between ages (P < 0.05).

The mRNA expression of Notch target genes *HES1, HEY1* and *HEY2* in the mammary gland during pubertal development was also evaluated (Fig. 4). Both target genes *HEY1* and *HEY2* increased from week 30 (early puberty) to 40 (late puberty), whereas no significant variation of *HES1* mRNA levels were observed. mRNA levels of the Notch ligands *JAGGED1* and *DELTA1* also increased gradually with pubertal development (Fig. 5). *JAGGED1* increased from 20 to 30 weeks, and *DELTA1* was significantly different between 20 weeks and 70 weeks.

**Figure 6:**
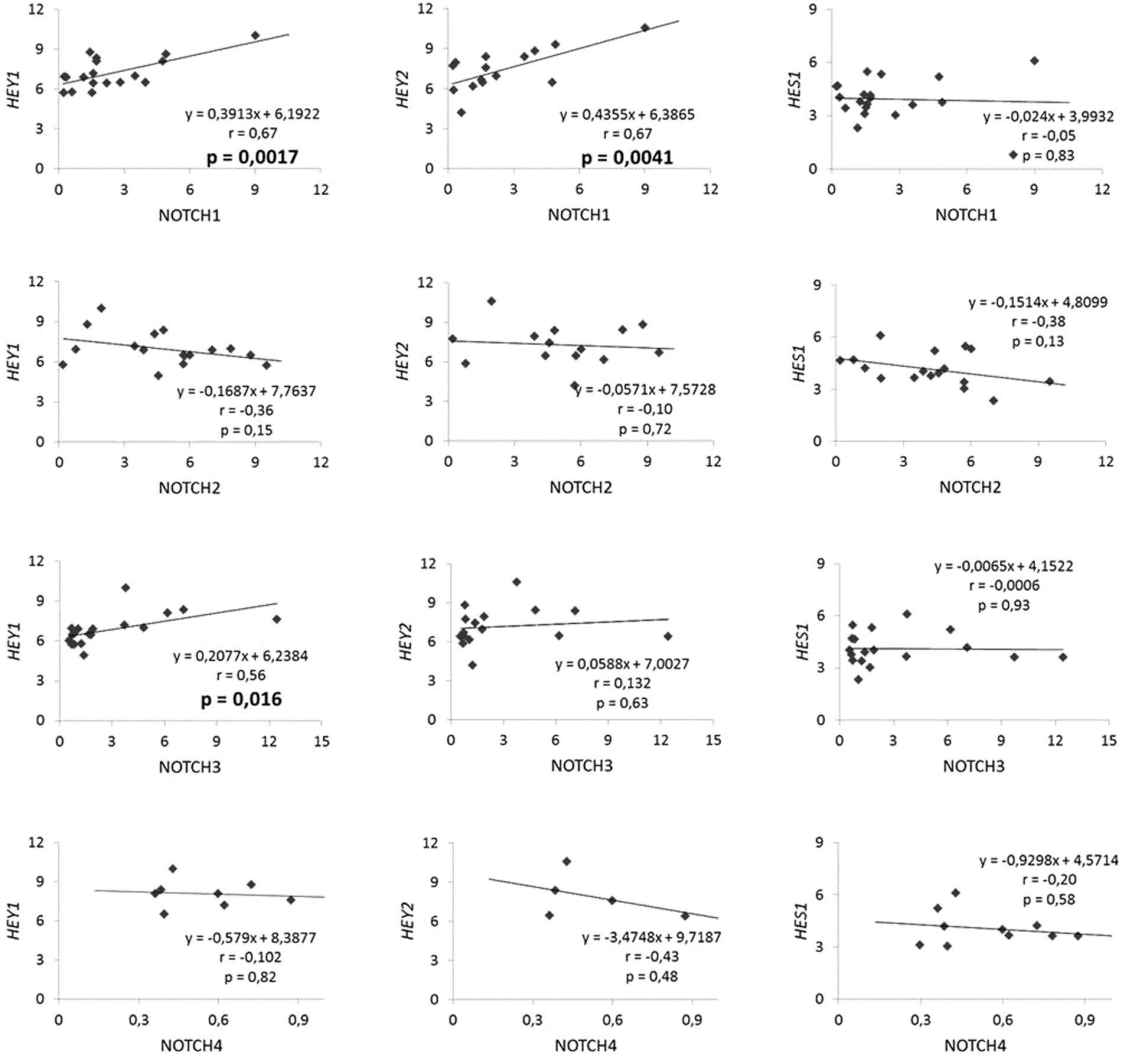
Correlations between NOTCH1–4 paralogues (NICD) and mRNA levels of *HEY1, HEY2* or *HES1* target genes. Each dot is the NICD relative to tubulin value for the NOTCH paralogue in the X axis and the mRNA level of the gene relative to GAPDH in the Y axis, of one heifer at one age. The equation of linear regression and coefficient of Pearson are shown in each graph. p ≤ 0.05 denotes a significant correlation.

Regression studies between active domains of the four Notch receptor paralogues and each studied target gene are shown in Fig. 6. NOTCH1 and NOTCH3 positively correlated with the target gene *HEY1* and NOTCH1 correlated with *HEY2*. NOTCH2 and NOTCH4 did not show correlation with any target gene studied. And *HES1* did not correlate with any Notch isoform.

## Discussion

The four Notch receptor paralogues were identified in the mammary gland of developing dairy heifers and their histological and subcellular localization described. A complete Notch signaling pathway was identified during pre and post-puberty in heifers, including receptors, ligands and target genes.

It is believed that individual receptors (NOTCH1–4) may play distinct roles in regulating mammary gland development by their cell-type specific distribution. For example, in humans a transcriptome analysis of cellular populations of the normal mammary gland showed that NOTCH1 and 3 were expressed mostly in luminal epithelial cells and NOTCH4 in myoepithelial cells^33,35^. In bovines, a recent transcriptomic analysis performed in pre-weaning heifers was able to detect activation of NOTCH1 in the mammary parenchyma^7^. In the present results, using immunohistochemistry, we identified specific patterns of Notch cellular and subcellular localization for each paralogue. Specific labeling for the four receptor paralogues in parenchymal cells (epithelial and myoepithelial) and stromal cells was evidenced with different intensities. In particular, stromal cells have been proposed to encompass mesenchymal stem cells, which will give rise to the lineages that form the stroma^10^, or already differentiated cells, such as endothelial cells or fibroblasts^13^. The NOTCH4 paralogue showed the highest label in myoepithelial cells, in accordance to the NOTCH4 restriction to the basal compartment described in human mammary cells^36^, and which has been related to mammary stem cell population^35^. All receptors were found predominantly at membrane and cytoplasmic levels, except NOTCH4, which was predominantly nuclear suggesting differential activation of this paralogue during mammary pubertal development. This is in accordance to suggested predominant role of NOTCH4 signaling in normal mammary gland development, participating in cell-fate determination^35^.

When developmental patterns of the four paralogues were analyzed by Western blot, we observed some differences in the higher weight 110 KDa band, which corresponds to the membrane receptor (MD)^37^, and in the lower weight 80 (or 72) KDa band, which corresponds to the active NICD clivated domain, for NOTCH1, 3 and 4. However, for NOTCH2 both band intensities followed similar patterns during development. As both bands are two forms of the same receptor, and the lower weight band represents the active form (NICD), clivated from the first one, and which will be translocated to the nucleus to exert its function by binding to DNA specific regulatory sites^38^, we analyzed the developmental pattern of the relative proportion of the NICD domain to (NICD + MD). This showed that the proportion of active NOTCH1 and NOTCH2 receptors increased during development, while a decrease with age was observed for active NOTCH3 and 4. The proportion of active NOTCH1 began to increase at early puberty (30 weeks of age), and was maximal at 40 weeks whereas active NOTCH2 increased at 40 weeks of age. On the other hand the relative proportion of NICD for NOTCH3 was high during all the prepubertal and pubertal period and decreased postpubertally. Enhanced NICD proportion at 30 and/or 40 weeks of age evidenced for these three paralogues, coincides with high cell division rate demonstrated at these ages by PCNA immunostaining^39^. At this time, around the onset of puberty, mammary gland parenchyma undergoes active growth in response to high estrogen concentration^3,40^, coinciding with increasing estradiol receptor alpha expression in the mammary gland^39,41^, and increasing circulating estradiol and IGF1 levels^42,43^. To this respect, Notch signaling pattern has been related to estradiol receptor regulation in breast cancer^44,45^ and in developmental studies in mouse^46^. Particularly, it has been proposed to control the differentiation of adipocytes and the development and remodeling of the vascular system, two critical components involved in the regulation of the development of the mammary gland^47^. With respect to NOTCH4, the proportion of its active form followed an opposite pattern during development, being maximal at 20 weeks of age (prepubertally), and then minimal, during puberty and postpuberty, suggesting an earlier activation of this paralogue. This may be in accordance to the current knowledge that NOTCH4 has a predominant role in stem cell maintenance and cell fate determination^35^, indicating a different role of this paralogue in mammary gland development. Overexpression of NOTCH inhibits cell differentiation during embryogenesis^25^, and therefore the decrease in NOTCH4 during development may indicate its role in allowing a correct cell differentiation in this tissue.

Different Notch receptors have partially different expression patterns in most tissues and at different stages of development. Moreover, specificity of the system within each tissue is reinforced by the diversity in the downstream targets activated by the NICD within each tissue. It is therefore paramount to study receptors, ligands and target genes^48^. The NICD binds to the RBPJ DNA binding protein to generate a transcription activating complex containing transcriptional coactivators. The formation of this complex results in the activation of several target genes, such as genes from the *HES/HEY* family, involved in cell growth, differentiation and survival^49^. In the present work, the expression of *HEY1* and *HEY2* increased in the mammary glands between 30 and 40 weeks of age, while no modification was observed for *HES1*, the other target gene studied. This suggests differential gene activation during advanced puberty, and a possible relation to the increase in NICD1 and the ligands *JAGGED1* and *DELTA1* observed during development. Furthermore, the significant correlation found between NICD1 with these two targets genes probably points to the specific activation of *HEY1 and 2* by NICD1 in mammary tissue. In the mouse mammary gland, the HEY/HES family genes were studied postpubertally and were differentially expressed during pregnancy, lactation and involution stages, suggesting differential involvement in mammary cycle regulation^34^. No studies have been conducted yet in the prepubertal gland. However, the fact that Notch components were affected by castration^34^, suggested that estrogen, which strongly increases just before puberty, may modulate the pathway. This is also coincident with the developmental increase in the mRNA expression of Notch ligands observed here. Cross-talk between NOTCH and estrogen receptors had also been reported in breast cancer studies^50^, suggesting further interaction in this tissue.

Although the activation of the target genes may be the result of the activation of all the different Notch paralogues, the one-to-one regression studies points to the receptors with greatest participation in the activation of each gene in this tissue. In this case we can suggest that NOTCH1 and NOTCH3 are preponderant in the activation of *HEY1* and *HEY2* and that NOTCH2 and 4 would have less participation, at least in the target genes evaluated herein. *HES1* expression, for which we did not find correlation with any of the four Notch paralogues, may be modulated by Notch receptors and/or their interaction with other molecules as suggested by other authors^51,52^. These results provide new evidence about the involvement of the Notch system in development of the bovine mammary gland. Importantly, the Notch system defines the context in which other pathways function to control proliferation, differentiation and cell survival, all processes which are critical for normal development and function of the mammary gland. Understanding the complete system, membrane receptors and ligands, as well as target genes, will bring light to the fine tuning of this orchestrated process.

## Materials and Methods

### Animals

Heifers were raised at the dairy farm of the Experimental School of Inchausti, 25 de Mayo, Province of Buenos Aires, Argentina (35°36′S, 60°32′W). At birth, female Holstein calves were placed in individual mobile pens, directly on the pastures for the first 60 days of life. The pens were moved when the floor became dirty or humid. They were fed 2 L of warm milk twice a day and had *ad libitum* access to balanced supplement (TDN 70%, CP 17%, fat 4%, non-protein N 0%, P 0.85%, Ca 1.2%, raw fiber 8%). At 2 months of age they were included in the grazing herd and grazed on alfalfa and (or) ryegrass pastures, in a rotational grazing system (stocking density: 8 animals/ha), with *ad libitum* access to the supplement. At 160 kg of BW, the supplement was changed to corn (2 kg/animal per day) and the stocking density was reduced to 2 animals/ha. Ten heifers were randomly chosen from the herd to obtain serial mammary biopsies at different ages. Each heifer was biopsied at each age. The ages were chosen so that there would be one age clearly pre-pubertal (20 weeks, 151 ± 4 Kg of body weight (BW)), two ages around the onset of puberty (30 and 40 weeks, 217 ± 5 and 261 ± 6 Kg BW, respectively), and one age post-pubertal (70 weeks, 371 ± 9 Kg BW)^53^. Briefly, heifers were sedated with 1% acepromazine (0.15 mg/Kg, Holliday Scott S.A., Buenos Aires, Argentina) and immobilized in a supine position. Udders were cleaned with soap and water, then rinsed and disinfected with iodinated solution. The biopsy was taken 2 cm away from the nipple, under local anesthesia (5 mL of lidocaine 2% sc)^40^. Each biopsy (at 20, 30, 40, and 70 wk) was obtained in a different place avoiding previous cicatrix. A Tru-Core I fully automatic biopsy gun provided with a Tru-Core 14 gauge × 20 cm needle (Medical Device Technologies Inc., Gainesville, FL) was used. The needle directly pierced the skin and took a gland sample 3-mm wide × 1-cm long at approximately 2.5 cm of depth with minimal lesion. Mammary gland samples were divided in three portions and one of them immediately placed in 4% formaldehyde for immunohistochemical studies, one in Tris-EDTA buffer (60 mM Tris-HCl, 1 mM EDTA pH 6.6 and a mix of proteases inhibitors) for Western blot analysis, and the last one in RNAlater® (Sigma-Aldrich Inc., St. Louis, MO) for mRNA quantification. The last two samples were stored at −70 °C until used. Only the samples with histologically verified mammary gland structure were used, and when the samples were not large enough, the portion destined to western blot processes was privileged. All the procedures were consistent with the Guide for the Care and Use of Agricultural Animals in Research and Teaching (FASS, 2010), and were approved by the ethics committee of the Institute of Biology and Experimental Medicine (N°CE058-2015).

### Immunohistochemistry

The portions of mammary biopsies fixed in 4% formaldehyde were transferred 24 h later to 70% ethanol until processed. Samples were rinsed abundantly with tap water and PBS (pH 7.4; 0.01 M), subjected to dehydration in increasing concentrations of ethanol, washed in xylol, and embedded in paraffin. Serial sections, 4 μm thick were cut with a manual microtome and mounted on glass slides previously coated with 3-aminopropyltriethoxysilane (Sigma-Aldrich Inc., St. Louis, MO). They were then deparaffinized, rehydrated and washed in PBS. Antigen retrieval was performed with sodium citrate 10 mM in microwave. Endogenous peroxidase activity was blocked with 3% H_2_O_2_ in PBS for 30 min at room temperature. Slides were preincubated in 3% egg albumin in PBS for 1 h to block nonspecific binding sites, and a solution of the first antibody was then added to the sections. The primary antibodies used were anti-NOTCH1 (1:200, sc-6014, Santa Cruz Biotechnology Inc., Dallas, TX), anti-NOTCH2 (1:200, 07-1234, Millipore Corp., Burlington MA), anti-NOTCH3 (1:200, sc-5593, Santa Cruz Biotechnology Inc., Dallas, TX) and anti-NOTCH4 (1:1000, A8303, NeoBioLab-NeoScientific, Cambridge, MA). After incubation overnight at 4 °C, slides were washed with PBS and incubated at room temperature for 1.5 h with a biotin-labeled anti-rabbit IgG secondary antibody (1:200, sc-2089, Santa Cruz Biotechnology Inc., Dallas, TX) and then with a preformed ABC complex for 30 min (Vectastain ABC kit; Vector Laboratories, Burlingame, CA). Subsequently, slides were immersed in a 0.05% 3,3-diaminobenzidine (Sigma-Aldrich Inc., St. Louis, MO) solution in PBS containing 0.01% H_2_O_2_. After brown color developed, the reaction was stopped by immersion in water, and sections were counterstained with hematoxylin, dehydrated, and coverslipped with DPX (Sigma-Aldrich Inc., St. Louis, MO). Immunoreactive cells were visualized with a PrimoStar microscope equipped with an Axiocam ERc 5 s digital camera (Zeiss, Obercochen, Germany) at a magnification of 40X.

### Western blot

Mammary biopsies stored in Tris-EDTA were washed with PBS and then homogenized in 150 µl ice-cold lysis buffer containing 50 mM HEPES, 140 mM NaCl, 1 mM EDTA, 10% glycerol, 1% Triton, 1 mM Na_3_VO_4_, 100 mM NaF, 1 mM PMSF and a mix of protease inhibitors in a handheld microtissue homogenizer. The homogenate was then centrifuged at 10,800 × g for 30 min at 4 °C. The pellet was discarded and an aliquot of supernatant was taken to quantify proteins by the Bradford method^54^. Samples were stored at −80 °C for subsequent Western blotting.

Fifty micrograms of proteins were mixed with 5X loading buffer. Samples were sonicated and heated 5 min at 95 °C and subjected to 10% SDS-PAGE. The gel was then blotted onto a nitrocellulose membrane (Amersham Hybond, GE), blocked with 2% bovine serum albumin in PBS-0.05% Tween and probed with the corresponding primary antibody followed by a secondary antibody conjugated with horseradish peroxidase. The primary antibodies used for these studies were NOTCH1 (1:1000, sc-6014, Santa Cruz Biotechnology Inc., Dallas, TX), NOTCH2 (1:1000, 07-1234, Millipore Corp., Burlington, MA), NOTCH3 (1:1000, sc-5593, Santa Cruz Biotechnology Inc., Dallas TX), NOTCH4 (1:5000, A8303, NeoBioLab-NeoScientific, Cambridge, MA) and Tubulin (1:70000, T-0198, Sigma-Aldrich, St. Louis, MO), as internal control. Secondary antibodies were donkey anti-rabbit (1:2000, sc-2089, Santa Cruz Biotechnology Inc., Dallas TX) and donkey anti-mouse (1:2000, sc-2098, Santa Cruz Biotechnology Inc., Dallas TX). Band intensities were detected by electrochemiluminescence (ImageQuant^TM^ LAS 4000, GE Healthcare, Uppsala, Sweden) and quantified using the ImageJ software.

### qRT-PCR

Total RNA was isolated from mammary biopsies stored in RNAlater® (Sigma-Aldrich Inc., St.Louis, MO) using TRI REAGENT (Molecular Research Center Inc., Cincinnati, OH). Each sample was homogenized in 200 µl TRI REAGENT and incubated at 30 °C for 5 min. Chloroform (40 µl) was added, samples were shaken vigorously, and after 5 min of incubation at 30 °C, they were centrifuged at 12000 × g for 15 min at 4 °C. Isopropanol (100 µl) was added to the supernatant to precipitate the RNA. After a 10-min incubation at 30 °C, samples were centrifuged at 12,000 × g for 10 min at 4 °C, supernatants discarded, and their pellets washed with 200 µl of 70% ethanol. Then samples were centrifuged at 7500 × g for 5 min at 4 °C and supernatants discarded. The resulting precipitates were resuspended in 5 µl free RNAses water, incubated for 10 min at 60 °C and then for 5 min on ice. RNA was quantified by Picodrop (Picodrop Ltd., Saffron Walden, United Kingdom) and the concentration was determined on the basis of absorbance at 260 nm, its purity was evaluated by the ratio of absorbance at 260/280 nm (~2.0 was considered appropriate), and its integrity was evaluated by agarose gel electrophoresis.

Total RNA (2 µg) was reversely transcribed in a reaction mixture containing 0.1 M dithiothreitol, 10 mM deoxynucleotide triphosphates, 5X RT Buffer, Oligo DT, Moloney murine leukemia virus transcriptase (Invitrogen, Life Technologies Corp., Carlsbad CA) and free RNAse water in a final volume of 20 µl. The reverse transcription polymerase chain reaction was performed in a Veriti end-point thermocycler (Applied Biosystems Inc., Foster City CA) with the following thermal cycling parameters: 10 minutes at 25 °C, 50 minutes at 37 °C and then held at 4 °C.

The product was amplified in a Real time thermocycler (LineGene 9600, Bioer, Hangzhou, China) with HES1, HEY1, HEY2, JAGGED1, DELTA1 and glycerol-3-phosphate dehydrogenase (GAPDH) sense and antisense primers (Supplementary Table S2) in a reaction mixture (15 µl) containing 0.5 µM of the primers (Integrated DNA Technologies, Coralville, IA), FastStart™ PCR Master Mix (Roche- Sigma Aldrich, Darmstadt, Germany) and free RNAse water. The expression levels of HES1, HEY1 and HEY2, DELTA1 and JAGGED1 mRNAs were determined using the comparative Ct method relative to the expression of housekeeping GAPDH.

### Statistical analysis

Results are expressed as means ± SEM. Each heifer was sampled at 20, 30, 40 and 70 days of age, therefore differences between time points were analyzed by one-way ANOVA with a repeated-measures design. Data were mathematically transformed to suit normalization if necessary. Post-hoc Tukey’s test was employed when necessary to define age differences between means for each gene or protein. Correlations between each Notch receptor and *HES1, HEY1* and *HEY2* expression were tested by Pearson parametric test. P < 0.05 was considered significant

### Ethical approval

All the procedures were consistent with the Guide for the Care and Use of Agricultural Animals in Research and Teaching (FASS, 2010), and were approved by the ethics committee of the Institute of Biology and Experimental Medicine (N°CE058-2015).

## Supplementary information

Supplementay information

## Data Availability

The datasets generated during and/or analyzed during the current study are available from the corresponding author on reasonable request.

## References

[1] Geiger AJ, Parsons CLM, Akers RM (2016). Feeding a higher plane of nutrition and providing exogenous estrogen increases mammary gland development in Holstein heifer calves. J Dairy Sci.

[2] Geiger AJ, Parsons CLM, Akers RM (2017). Feeding an enhanced diet to Holstein heifers during the preweaning period alters steroid receptor expression and increases cellular proliferation. J Dairy Sci.

[3] Akers RM, Ellis SE, Berry SD (2005). Ovarian and IGF-I axis control of mammary development in prepubertal heifers. Domestic animal endocrinology.

[4] Hovey RC, Trott JF (2004). Morphogenesis of mammary gland development. Advances in experimental medicine and biology.

[5] Akers RM (2006). Major advances associated with hormone and growth factor regulation of mammary growth and lactation in dairy cows. Journal of dairy science.

[6] Soberon F, Van Amburgh ME (2017). Effects of preweaning nutrient intake in the developing mammary parenchymal tissue. J Dairy Sci.

[7] Vailati-Riboni M (2018). Higher plane of nutrition pre-weaning enhances Holstein calf mammary gland development through alterations in the parenchyma and fat pad transcriptome. BMC genomics.

[8] Hare KS (2019). Preweaning nutrient supply alters mammary gland transcriptome expression relating to morphology, lipid accumulation, DNA synthesis, and RNA expression in Holstein heifer calves. J Dairy Sci.

[9] Shackleton M (2006). Generation of a functional mammary gland from a single stem cell. Nature.

[10] Capuco AV, Choudhary RK, Daniels KM, Li RW, Evock-Clover CM (2012). Bovine mammary stem cells: cell biology meets production agriculture. Animal: an international journal of animal bioscience.

[11] Borena BM, Bussche L, Burvenich C, Duchateau L, Van de Walle GR (2013). Mammary stem cell research in veterinary science: an update. Stem cells and development.

[12] Arendt LM, Kuperwasser C (2015). Form and function: how estrogen and progesterone regulate the mammary epithelial hierarchy. Journal of mammary gland biology and neoplasia.

[13] Rauner G, Barash I (2012). Cell hierarchy and lineage commitment in the bovine mammary gland. PloS one.

[14] Perruchot MH (2016). Mammary Epithelial Cell Hierarchy in the Dairy Cow Throughout Lactation. Stem cells and development.

[15] Collu GM, Hidalgo-Sastre A, Brennan K (2014). Wnt-Notch signalling crosstalk in development and disease. Cellular and molecular life sciences: CMLS.

[16] Sjöqvist Marika, Andersson Emma R. (2019). Do as I say, Not(ch) as I do: Lateral control of cell fate. Developmental Biology.

[17] Blaumueller CM, Qi H, Zagouras P, Artavanis-Tsakonas S (1997). Intracellular cleavage of Notch leads to a heterodimeric receptor on the plasma membrane. Cell.

[18] Kopan R. (2012). Notch Signaling. Cold Spring Harbor Perspectives in Biology.

[19] Bray SJ (2006). Notch signalling: a simple pathway becomes complex. Nature reviews. Molecular cell biology.

[20] Kopan R, Ilagan MX (2009). The canonical Notch signaling pathway: unfolding the activation mechanism. Cell.

[21] Bray SJ (2016). Notch signalling in context. Nature reviews. Molecular cell biology.

[22] Ranganathan P, Weaver KL, Capobianco AJ (2011). Notch signalling in solid tumours: a little bit of everything but not all the time. Nature reviews. Cancer.

[23] Gude N, Sussman M (2012). Notch signaling and cardiac repair. Journal of molecular and cellular cardiology.

[24] Sawada M, Sawamoto K (2013). Mechanisms of neurogenesis in the normal and injured adult brain. The Keio journal of medicine.

[25] Koch U, Lehal R, Radtke F (2013). Stem cells living with a Notch. Development.

[26] Faigle R, Song H (2013). Signaling mechanisms regulating adult neural stem cells and neurogenesis. Biochimica et biophysica acta.

[27] Chen S, Lee BH, Bae Y (2014). Notch signaling in skeletal stem cells. Calcified tissue international.

[28] Fre S (2005). Notch signals control the fate of immature progenitor cells in the intestine. Nature.

[29] Sale S, Lafkas D, Artavanis-Tsakonas S (2013). Notch2 genetic fate mapping reveals two previously unrecognized mammary epithelial lineages. Nature cell biology.

[30] Dontu G (2004). Role of Notch signaling in cell-fate determination of human mammary stem/progenitor cells. Breast cancer research: BCR.

[31] Rangel MC (2016). Developmental signaling pathways regulating mammary stem cells and contributing to the etiology of triple-negative breast cancer. Breast cancer research and treatment.

[32] Kapila N (2016). Impact of Heat Stress on Cellular and Transcriptional Adaptation of Mammary Epithelial Cells in Riverine Buffalo (Bubalus Bubalis). PloS one.

[33] Raouf A (2008). Transcriptome analysis of the normal human mammary cell commitment and differentiation process. Cell stem cell.

[34] Raafat A (2011). Expression of Notch receptors, ligands, and target genes during development of the mouse mammary gland. Journal of cellular physiology.

[35] Malhotra GK, Zhao X, Band H, Band V (2011). Shared signaling pathways in normal and breast cancer stem cells. Journal of carcinogenesis.

[36] Harrison H (2010). Regulation of breast cancer stem cell activity by signaling through the Notch4 receptor. Cancer research.

[37] Perrone S (2017). Notch system is differentially expressed and activated in pituitary adenomas of distinct histotype, tumor cell lines and normal pituitaries. Oncotarget.

[38] Mumm JS, Kopan R (2000). Notch signaling: from the outside. Developmental biology.

[39] Perri AF (2014). Cellular proliferation rate and insulin-like growth factor binding protein (IGFBP)-2 and IGFBP-3 and estradiol receptor alpha expression in the mammary gland of dairy heifers naturally infected with gastrointestinal nematodes during development. J Dairy Sci.

[40] Perri AF (2013). Gastrointestinal parasite control during prepuberty improves mammary parenchyma development in Holstein heifers. Veterinary parasitology.

[41] Mueller SO, Clark JA, Myers PH, Korach KS (2002). Mammary gland development in adult mice requires epithelial and stromal estrogen receptor alpha. Endocrinology.

[42] Lacau-Mengido IM (2000). Endocrine studies in ivermectin-treated heifers from birth to puberty. J Anim Sci.

[43] Chandrashekar V, Bartke A (2003). The role of insulin-like growth factor-I in neuroendocrine function and the consequent effects on sexual maturation: inferences from animal models. Reproductive biology.

[44] Rizzo P (2008). Cross-talk between notch and the estrogen receptor in breast cancer suggests novel therapeutic approaches. Cancer research.

[45] Hao L (2010). Notch-1 activates estrogen receptor-alpha-dependent transcription via IKKalpha in breast cancer cells. Oncogene.

[46] Huang B (2016). Dysregulation of Notch and ERalpha signaling in AhR-/- male mice. Proceedings of the National Academy of Sciences of the United States of America.

[47] Callahan R, Egan SE (2004). Notch signaling in mammary development and oncogenesis. Journal of mammary gland biology and neoplasia.

[48] Andersson ER, Sandberg R, Lendahl U (2011). Notch signaling: simplicity in design, versatility in function. Development.

[49] Yamaguchi N (2008). NOTCH3 signaling pathway plays crucial roles in the proliferation of ErbB2-negative human breast cancer cells. Cancer research.

[50] Rizzo P (2009). Targeting Notch signaling cross-talk with estrogen receptor and ErbB-2 in breast cancer. Advances in enzyme regulation.

[51] Iso T, Kedes L, Hamamori Y (2003). HES and HERP families: multiple effectors of the Notch signaling pathway. Journal of cellular physiology.

[52] Fischer A, Gessler M (2007). Delta-Notch–and then? Protein interactions and proposed modes of repression by Hes and Hey bHLH factors. Nucleic acids research.

[53] Mejia M (1999). Effects of continuous ivermectin treatment from birth to puberty on growth and reproduction in dairy heifers. J Anim Sci.

[54] Bradford MM (1976). A rapid and sensitive method for the quantitation of microgram quantities of protein utilizing the principle of protein-dye binding. Analytical biochemistry.

