# Supplementary material for: The Notch system during pubertal development of the bovine mammary gland
Source: Sci Rep. 2019 Jun 20;9:8899. doi: 10.1038/s41598-019-45406-6 (PMC6586787; doi:10.1038/s41598-019-45406-6)
Supplement: Supplementary file 1 — Supplementay information [file 41598_2019_45406_MOESM1_ESM.pdf]

Supplementary Information

The Notch system during pubertal development of the bovine mammary gland.

Nadia Bonadeo[1], Damasia Becu-Villalobos[2], Carolina Cristina[1], Isabel M. Lacau-Mengido[2*]

1- Centro de Investigaciones Básicas y Aplicadas, Centro de Investigaciones y Transferencia del Noroeste de la Provincia de Buenos Aires, Monteagudo 2772, Pergamino 2700, Buenos Aires, Argentina

2- Instituto de Biología y Medicina Experimental, Vuelta de Obligado 2490, CABA 1428, Argentina

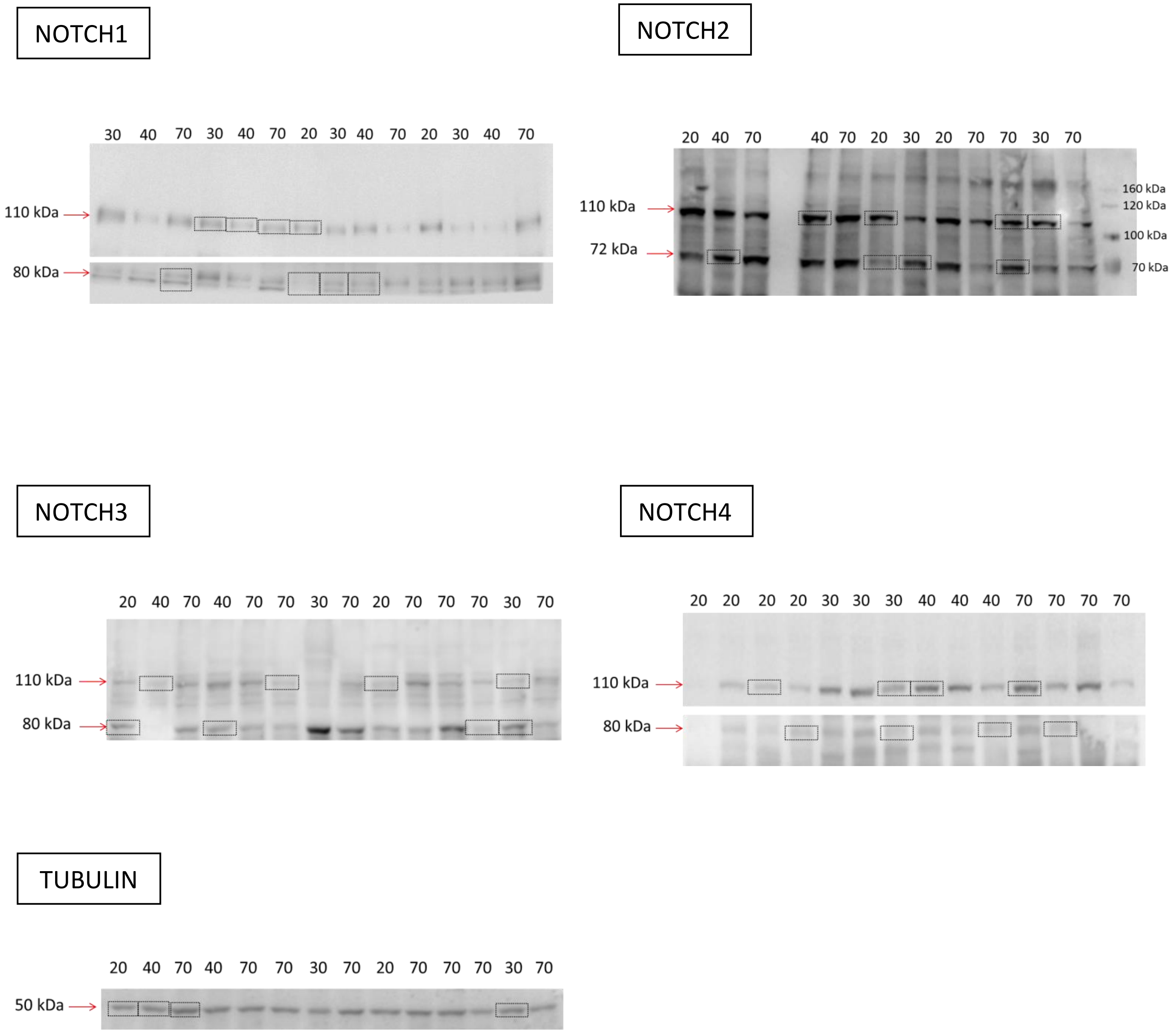

Supplementary Figure S1. Representative full-length Western blots showing the active (80 or 72 kDa) and membrane domains (110 kDa) of NOTCH1-4 receptors and tubulin (50 kDa) in the four ages studied. Boxes identify the bands chosen for figure 2e.

Primers	Sequences	Annealing temperature
HES1	Fo: 5´-AGTTCGCGGCATTCCAAGC-3´<br>Re: 5´-CCTCGTTCATGCACTCGCT-3´	60,9°C
HEY1	Fo: 5´-AGACGGAGAGGCATTATTGAGAAG-3´<br>Re: 5´-GATAGTCCATAGCAAGGGCGT-3´	59,9°C
HEY2	Fo: 5´-TCTGAGTTGAGACGACTGGTG-3´<br>Re: 5´-GTGCATCAAAGTAGCCTTTACCC-3´	59,6°C
JAGGED1	Fo: 5´-TGCAGTCCTCATCCCTGTTAC-3´<br>Re: 5´-TGATCTCATCCACGCAGGTC-3´	59,5°C
DELTA1	Fo: 5´-TTTGGACACTTCACCTGCGG-3´<br>Re: 5´-TGCAGTAGTTGAGGTCCTGGT-3´	60,8°C
GAPDH	Fo: 5´-CACTGTCCACGCCATCACT-3´<br>Re: 5´-GCCTGCTTCACCACCTTCT-3´	60,0°C

Supplementary Table S2. Sequences and annealing temperatures of primers used.
